# Biophysical Controls on Light Response of Net CO_2_ Exchange in a Winter Wheat Field in the North China Plain

**DOI:** 10.1371/journal.pone.0089469

**Published:** 2014-02-20

**Authors:** Xiaojuan Tong, Jun Li, Qiang Yu, Zhonghui Lin

**Affiliations:** 1 College of Forestry, Beijing Forestry University, Beijing, China; 2 Key Laboratory of Water Cycle and Related Land Surface Processes, Institute of Geographic Science and Natural Resources Research, Chinese Academy of Sciences, Beijing, China; 3 Plant Functional Biology and Climate Change Cluster, University of Technology, Sydney, Australia; DOE Pacific Northwest National Laboratory, United States of America

## Abstract

To investigate the impacts of biophysical factors on light response of net ecosystem exchange (NEE), CO_2_ flux was measured using the eddy covariance technique in a winter wheat field in the North China Plain from 2003 to 2006. A rectangular hyperbolic function was used to describe NEE light response. Maximum photosynthetic capacity (*P*_max_) was 46.6±4.0 µmol CO_2_ m^−2^ s^−1^ and initial light use efficiency (α) 0.059±0.006 µmol µmol^−1^ in April−May, two or three times as high as those in March. Stepwise multiple linear regressions showed that *P*_max_ increased with the increase in leaf area index (LAI), canopy conductance (*g*_c_) and air temperature (*T*_a_) but declined with increasing vapor pressure deficit (VPD) (*P*<0.001). The factors influencing *P*_max_ were sorted as LAI, *g*_c_, *T*_a_ and VPD. α was proportional to ln(LAI), *g*_c_, *T*_a_ and VPD (*P*<0.001). The effects of LAI, *g*_c_ and *T*_a_ on α were larger than that of VPD. When *T*_a_>25°C or VPD>1.1−1.3 kPa, NEE residual increased with the increase in *T*_a_ and VPD (*P*<0.001), indicating that temperature and water stress occurred. When *g*_c_ was more than 14 mm s^−1^ in March and May and 26 mm s^−1^ in April, the NEE residuals decline disappeared, or even turned into an increase in *g*_c_ (*P*<0.01), implying shifts from stomatal limitation to non-stomatal limitation on NEE. Although the differences between sunny and cloudy sky conditions were unremarkable for light response parameters, simulated net CO_2_ uptake under the same radiation intensity averaged 18% higher in cloudy days than in sunny days during the year 2003−2006. It is necessary to include these effects in relevant carbon cycle models to improve our estimation of carbon balance at regional and global scales.

## Introduction

Vegetation productivity is the foundation of carbon sequestration and grain yield formation. For the limit on field observation techniques, early studies mainly focused on photosynthesis at the leaf level. The biochemistry and ecophysiology of leaf photosynthesis have been well understood and parameterized [1], [2]. However, it is difficult to measure leaf photosynthesis over long periods and upscale photosynthetic rate from the leaf level to the canopy or ecosystem level because of the non-linear distribution of leaf area and radiation intensity within the vegetation canopy. With the development of micrometeorological techniques, especially the eddy covariance (EC) method, net photosynthetic rate of vegetation could be directly measured at the ecosystem level. The EC technique has been widely used in CO_2_ flux measurements in forest, grassland and farmland ecosystems [3]–[9].

Plant photosynthesis is primarily driven by incident solar radiation. Light response models, including rectangular hyperbolic model [10], [11] and non-rectangular hyperbolic model [12], [13], were developed to describe the relationship between daytime net ecosystem CO_2_ exchange (NEE) and photosynthetically active radiation (PAR). Besides solar radiation, factors influencing daytime NEE include environmental variables such as air temperature, vapor pressure deficit and soil water content and biological variables such as leaf area index and canopy conductance. These factors may be considered by (1) estimating the bias of simulated NEE as functions of influencing factors [14], [15], or (2) revising light response parameters as functions of influencing factors [9], [11], [16]–[19]. The effects of biophysical factors on light response parameters are often estimated using stepwise multiple linear regression models [9]. However, these models have not been used to assess the influence of variables on NEE residual so far.

Long-term observations and simulations have shown a worldwide decrease in surface solar radiation (global dimming) from 1950s to 1980s, with a partial recovery (brightening) in 1990s in some areas (e.g. high and middle latitude of the Northern Hemisphere) [20]. During the dimming period, direct radiation declined remarkably, whereas diffuse radiation enhanced. The increase in diffuse fraction of surface solar radiation may be attributed to increasing aerosol and/or cloudiness, as both factors tend to enhance scattering in the atmosphere [20]. Light responses of photosynthesis under various sky conditions were investigated in forests [8], [21]–[23] and croplands [24], [25]. For the tall and dense vegetations, photosynthesis may be enhanced by diffuse radiation because diffuse light distributes more effectively within the canopy compared with direct light [26]. After considering the effects of direct and diffuse radiation on canopy photosynthesis in the model, Mercado et al. [27] found that the increases in diffuse fraction enhanced the global land carbon sink by 23.7% during the period from 1960 to 1999.

China is the largest wheat producer and consumer in the world [28] and continuously attempts to increase its production to ensure national food security. As one of large food production regions in China, the North China Plain (NCP) produces about half of the country's wheat [29]. In this study, CO_2_ flux was measured continuously using the EC technique in a winter wheat field in the North China Plain for 4 years. The objectives are to (1) investigate NEE light response and the influencing factors, and (2) assess the effects of sky conditions on NEE light response. This study will improve our knowledge on the parameterization of carbon cycle models and the scenario analyses of carbon sink in the future under changing climate.

## Materials and Methods

### Study site

This study was conducted at Yucheng Comprehensive Experiment Station, Chinese Academy of Sciences (36°57′N, 116°38′E, 23.4 m). It is located at the North China Plain, with a temperate monsoon climate. Mean annual temperature is 13.1°C and annual solar radiation is 5242 MJ m^−2^. Annual precipitation is about 528 mm. Soil organic content is 1.21% and pH value is about 7.9. The typical cropping system in this region is the biannual rotation with winter wheat and summer maize. In this study, winter wheat was planted in mid/late October and harvested in early/mid June. The detailed field management was described by Tong et al. [30].

### Field observations

CO_2_ and latent heat fluxes were measured by the eddy covariance system with a three-dimensional sonic anemometer (model CSAT3, Campbell Sci. Inc., USA) and an infrared open-path CO_2_/H_2_O gas analyzer (model LI-7500, Li-Cor Inc., USA). The eddy covariance system, mounted at the height of 2.1 m, was used to measure 3-D wind speed, air temperature, humidity and CO_2_ concentration above the canopy. Raw data were collected at 10 Hz and recorded by a CR5000 datalogger (model CR5000, Campbell Scientific Inc., USA). The CR5000 datalogger can store data and programs on a PC card. The card can be carried to a computer and the computer reads PC cards via its PCMCIA card slot directly.

Anemometers (model A100R, Vector, UK) and psychrometers (model HMP45C, Vaisala, Finland) were installed at heights of 2.05 and 3.25 m above the ground. Photosynthetically active radiation (PAR) was measured using a quantum sensor (model Li-190SB, Li-Cor Inc, USA). Solar radiation and net radiation (*R*_n_) were measure by a pyranometer (model CM11, Kipp & Zonen, Delft, The Netherlands) and a net radiometer (model CNR-1, Kipp & Zonen, Delft, The Netherlands), respectively. Two soil heat flux plates were buried in the depth of 2 cm, one between rows and another between plants. Soil temperature was measured at 0, 5, 10, 30 and 50 cm depths. Soil water content (SWC) at the depths of 20 cm and 30 cm was measured with time domain reflectometers (TDR) (model CS616, Campbell Sci. Inc., USA). Rainfall was measured with a rain gauge (model 52203, Rm Young MI, USA). All meteorological data were recorded with a data logger (model CR23x, Campbell Sci. Inc., USA) and were stored at intervals of 30 min.

Biomass, leaf area index (LAI) and plant height were measured every 5 days during the growing season of winter wheat. There were three sampling plots (replications) for each measurement. Twenty plants were sampled continuously for each plot and the plots were selected randomly. Leaf area was measured with Leaf area instrument (model Li-3100, Campbell Sci. Inc., USA). In this study, the main growing season began when the cropland turned from the carbon source to the sink (5-day moving mean CO_2_ flux from positive to negative), and it ended when shifted reversely.

### Flux data quality control

CO_2_ and latent heat fluxes were calculated as follows:
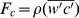
(1)




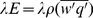
(2)where *ρ* is air density, *w*' the vertical wind velocity, *c*' CO_2_ concentration, *λ* the latent heat of vaporization, *E* water vapor flux and *q* the specific humidity. Overbars indicate an averaging operation and primes denote deviations from the mean.

Raw data were conducted by two dimension coordinate rotations [31] and Webb-Pearman-Leuning (WPL) correction [32] to obtain 30-min mean flux data. CO_2_ and water vapor fluxes could be affected by rain and dew. The abnormal data were eliminated following the method used by Falge et al. [33]. In the daytime, data gaps were 29%, 8%, 10% and 16% in the growing seasons of winter wheat in 2003, 2004, 2005 and 2006, respectively. Gap filled data were not used in this paper.

### Light response of NEE

In most ecosystems, the relationship between daytime NEE and PAR can be expressed by a rectangular hyperbolic function [33], [34]:
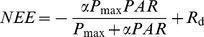
(3)where α is the initial slope of the light response curve (initial light use efficiency), *P*_max_ the maximum photosynthetic capacity, *R*_d_ the daytime ecosystem respiration rate under dark conditions. The model (Eq. (3)) was fitted by the software “Origin 7.0” (Microcal Software Inc.). In this study, negative NEE means net CO_2_ uptake by the cropland and positive NEE indicates net CO_2_ emission from the cropland.

To study the environmental factors influencing daytime NEE besides PAR, NEE was computed using Eq. (3) and the residual (NEE_r_) was obtained as measured NEE minus simulated NEE. Stepwise multiple linear regression models were used to estimate the integrative influences of biophysical factors on NEE_r_. Statistical analyses were performed using the software “SPSS 13.0” (SPSS Inc.). According to the method used by Carrara et al. [17], Powell et al. [18] and Teklemariam et al. [7], air temperature (*T*_a_), vapor pressure deficit (VPD), soil water content (SWC), leaf area index (LAI) and canopy conductance (*g*_c_) were divided into many classes and mean NEE_r_ was obtained for each class to show the trends in NEE_r_ versus variables.

To investigate seasonal patterns of light response parameters, a rectangular hyperbola (Eq. (3)) was used to describe the light response of NEE every five days and a time series of α, *P*_max_ and *R*_d_ were obtained. Meanwhile, the biophysical variables in the daytime with the 5-day interval were averaged. Stepwise multiple linear regression analyses were applied to distinguish the key drivers for α, *P*_max_ and *R*_d_. The relationships among light response parameters were also investigated at the 5-day scale.

Light response parameters varied among years. Standard errors (*e*_1_ and *e*_2_) were calculated for sunny and cloudy sky conditions and total standard error (*e*) was obtained for both sky conditions using the equation as follows:
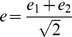
(4)


The difference between sunny and cloudy sky conditions would be pronounced if it was larger than the total standard error for two sky conditions.

### Canopy conductance

According to Monteith and Unsworth [35], canopy conductance (*g*_c_) was calculated as follows:
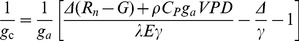
(5)where *λE* is the latent heat flux (W m^−2^), *R*_n_ net radiation (W m^−2^), *G* soil heat flux (W m^−2^), △ the slope of saturation vapor pressure to the air temperature curve (kPa K^−1^), *ρ* air density (mol m^−3^), *C*_p_ the specific heat capacity of air (J mol^−1^ K^−1^), VPD vapor pressure deficit (kPa), *γ* the psychrometric constant (kPa K^−1^), *g*_c_ canopy conductance (m s^−1^), and *g*_a_ aerodynamic conductance (m s^−1^) which can be given as follows [36]:
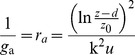
(6)where ra is aerodynamic resistance (s m−1), z the height where wind velocity measured (m), d zero-plane displacement height (m), z0 roughness length (m), k the von Karman constant (0.41) and u wind velocity (m s−1). z0 and d can be calculated as [37], [38]:



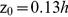
(7)

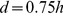
(8)where *h* is the crop height (m).

### Defining sunny and cloudy sky conditions

**Figure 1:**
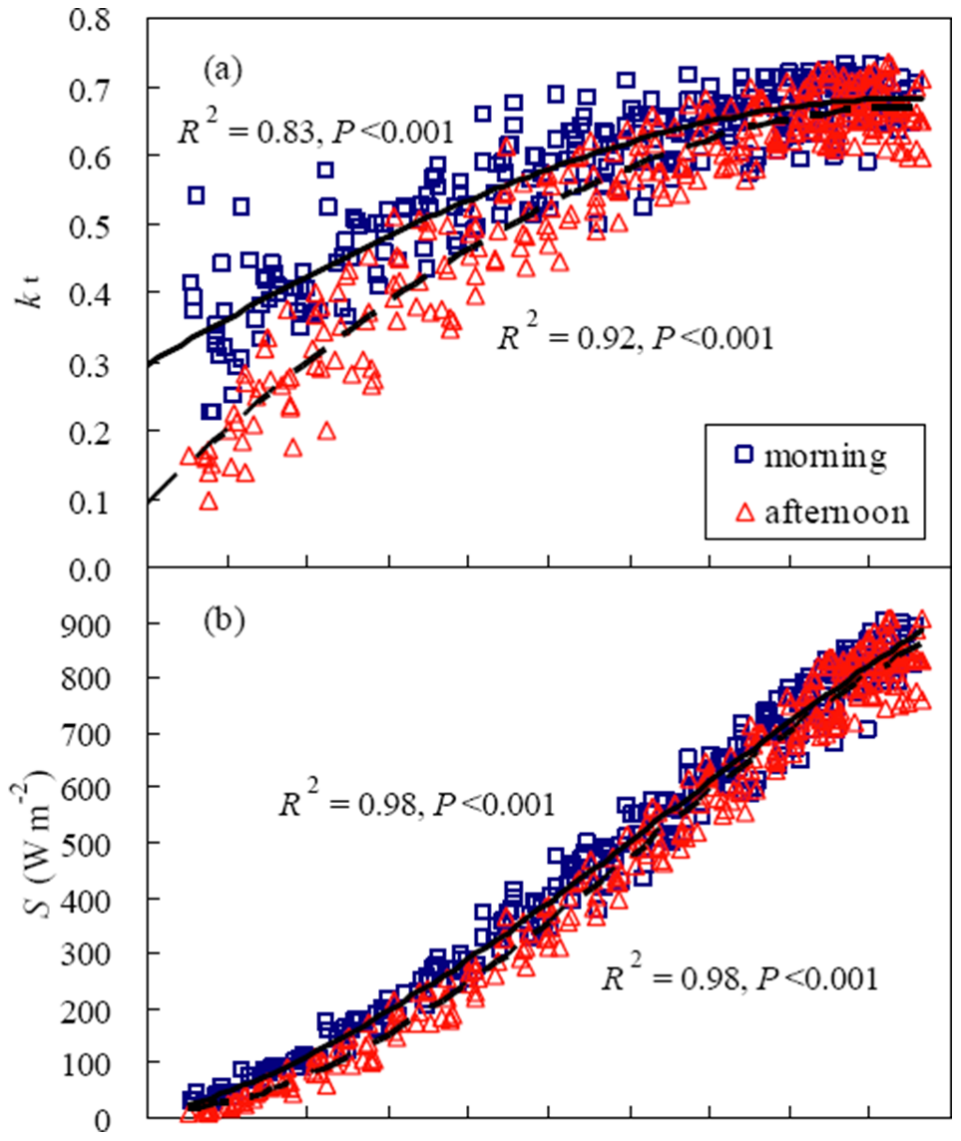
Scatter plots and regressions between (a) the clearness index (*k*_t_) and the sine of solar elevation angles (sin*β*), and (b) global solar radiation (*S*) and sin*β* for a winter wheat field in April-May 2003. The data were fitted by cubic polynomials in the morning (solid line) and afternoon (dashed line), respectively.

The clearness index (*k*_t_) is used to describe sky conditions and the degree of impact of cloudiness on the solar radiation received at the Earth's surface [22]. It can be given by [39]:
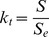
(9)




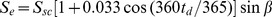
(10)




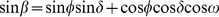
(11)where *S* is global solar radiation (W m^−2^), *S*_e_ the extraterrestrial irradiance at a plane parallel to the earth surface (W m^−2^), *S*_sc_ the solar constant (1370 W m^−2^), *t*_d_ the day of year, *β* the solar elevation angle, *φ* the local latitude, *δ* the declination of the sun, and *ω* hour angle. The sky conditions were classified at a half-day scale because the days without clouds for the whole daytime were rare [39]. In this study, sunny skies were simply identified when the half-day mean *k*_t_ was larger than a threshold in the morning and afternoon, respectively. 1.2 times of two-month running average *k*_t_ was used as the threshold so that the number of obtained sunny half-days was close to the mean value observed in recent decades. Clearness index were plotted against solar elevation angles and fitted by cubic polynomials in the sunny mornings and afternoons, respectively (Fig. 1). The data falling away from the major patterns were excluded [39]. Different from the studies in the forests [22], [39], *k*_t_ observed in the wheat field was lower in the afternoon than in the morning (Fig. 1a). Air pollution may reduce the atmospheric transparency, resulting in a small incident solar radiation at the land surface. The reduction was more evident in the afternoon than in the morning (Fig. 1b).

## Results

### NEE Light response and influencing factors

**Figure 2:**
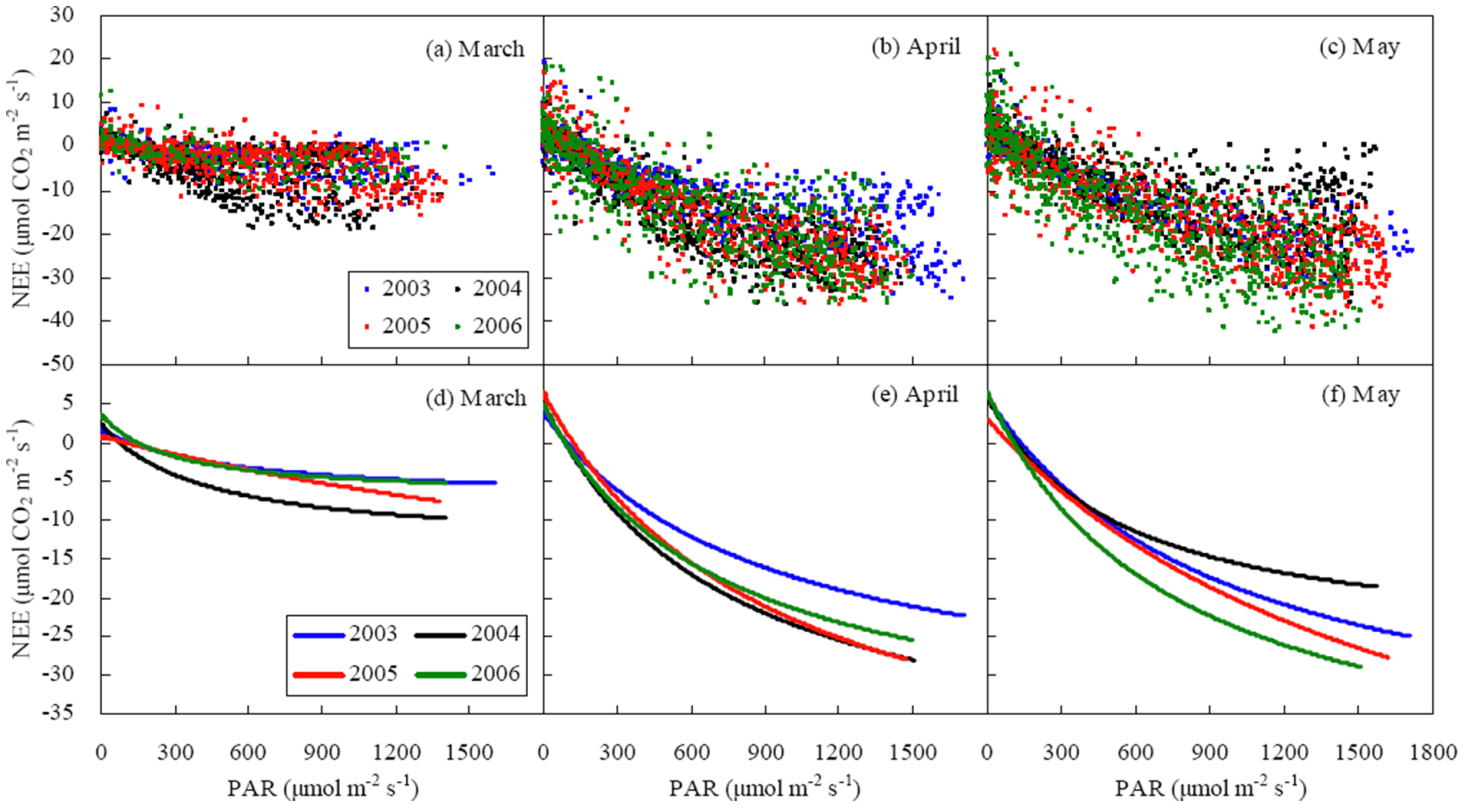
Response of net ecosystem CO_2_ exchange (NEE) to photosynthetically active radiation (PAR) in a winter wheat field. a–c: NEE obtained by eddy covariance technique; d–f: light response curves fitted monthly using the rectangular hyperbolic function (Eq. (3)). The values of light response parameters were shown in Table 1.

The relationship between daytime NEE and PAR in a winter wheat field is shown in Figure 2. Light response curves in April and May were similar but both of them differed from those in March. From 2003 to 2006, *P*_max_ was 46.6±4.0 µmol CO_2_ m^−2^ s^−1^, α 0.059±0.006 µmol µmol^−1^ and *R*_d_ 5.3±0.3 µmol CO_2_ m^−2^ s^−1^ in April−May, two or three times as high as those in March (Table 1). However, the inter-annual coefficients of variation (CV) for α, *P*_max_ and *R*_d_ were great in March due to large variations of LAI, daytime mean *T*_a_, VPD and SWC among years. The variations in VPD and SWC resulted from significant changes in precipitation in March among years (Tables 1 and 2). In March 2005, α was so small and *P*_max_ was so large that the light response curves were actually close to a line (Table 1 and Fig. 2).

**Table 1:** Monthly NEE light response parameters (*P*_max_, α and *R*_d_) derived from Eq. (3) for a winter wheat field.

Month	Year	*P*_max_(µmol CO_2_ m^−2^ s^−1^)	α(µmol µmol^−1^)	*R*_d_(µmol CO_2_ m^−2^ s^−1^)	*r* ^2^	*n*
March	2003	9.2	0.017	1.6	0.388^***^	497
	2004	15.8	0.037	2.4	0.396^***^	681
	2005	29.9	0.008	0.8	0.360^***^	701
	2006	10.6	0.036	3.5	0.431^***^	179
April	2003	40.1	0.044	3.8	0.736^***^	695
	2004	49.7	0.067	5.2	0.836^***^	746
	2005	56.4	0.060	6.4	0.806^***^	719
	2006	44.8	0.063	5.0	0.629^***^	692
May	2003	48.7	0.050	5.9	0.876^***^	402
	2004	32.8	0.061	5.9	0.619^***^	779
	2005	64.5	0.036	3.1	0.723^***^	743
	2006	53.4	0.070	6.5	0.715^***^	754

*P*_max_: maximum photosynthetic capacity;

α: initial light use efficiency;

*R*_d_: daytime ecosystem respiration under dark conditions.

Significance of the regression was “^***^” for *P*<0.001.

**Table 2:** Monthly biophysical variables in the winter wheat field from March to May in the year 2003–2006.

Month	Year	*T*_a_(°C)	VPD(kPa)	SWC(m^3^ m^−3^)	Prec(mm)	LAI
March	2003	8.7	0.57	0.184	42.5	0.61
	2004	10.5	0.69	0.136	55.7	1.47
	2005	7.9	0.65	0.100	0.1	1.07
	2006	10.4	0.81	0.106	0.2	0.86
April	2003	15.3	0.73	0.186	160.3	3.20
	2004	17.1	0.88	0.141	52.4	5.69
	2005	17.2	0.96	0.139	27.5	4.49
	2006	15.7	0.79	0.124	19.7	3.81
May	2003	21.9	0.99	0.160	12.4	2.99
	2004	21.1	1.10	0.134	46.8	3.44
	2005	21.1	1.07	0.145	37.0	4.61
	2006	20.9	0.96	0.138	62.4	4.95

*T*_a_: daytime mean air temperature;

VPD: daytime mean vapor pressure deficit;

SWC: daytime mean soil water content at a depth of 20 cm;

Prec: total precipitation;

LAI: mean leaf area index.

Table 3 indicates that NEE_r_ declined with the increases in LAI, *g*_c_, *T*_a_ and SWC (*P*<0.001) but increased with the increase in VPD (*P*<0.001). Positive/negative NEE_r_ means measured NEE was higher/lower than simulated NEE; or measured CO_2_ uptake was lower/higher than simulated CO_2_ uptake. The impacts of biophysical factors on NEE_r_ varied in different months. Factors influencing NEE_r_ were sorted as LAI, SWC, *g*_c_, *T*_a_ and VPD in March; *g*_c_, LAI, VPD and *T*_a_ in April; and LAI, *g*_c_, VPD and *T*_a_ in May. The effects of SWC on NEE_r_ were significant in March but insignificant in April and May (Table 3).

**Table 3:** Influences of biophysical factors (*T*_a_, VPD, SWC, *g*_c_ and LAI) on NEE residual in a winter wheat field from March to May in the year 2003–2006, estimated by the stepwise multiple linear regression models.

Month	Independent variables	*F*	*r* ^2^	Model
March	LAI	644.33^***^	0.289	NEE_r_ = −2.854LAI+2.813
	LAI, SWC	382.77^***^	0.326	NEE_r_ = −2.918LAI−17.883SWC+5.257
	LAI, SWC, *g*_c_	284.79^***^	0.350	NEE_r_ = −2.732LAI−16.507SWC−0.126*g*_c_+5.498
	LAI, SWC, *g*_c_, *T*_a_	215.08^***^	0.352	NEE_r_ = −2.611LAI−15.341SWC−0.127*g*_c_−0.029*T*_a_+5.496
	LAI, SWC, *g*_c_, *T*_a_, VPD	182.50^***^	0.366	NEE_r_ = −2.461LAI−10.812SWC−0.112*g*_c_−0.149*T*_a_+1.883VPD+4.499
April	*g*_c_	386.84^***^	0.125	NEE_r_ = −0.289*g*_c_+2.855
	*g*_c_, LAI	315.42^***^	0.188	NEE_r_ = −0.274*g*_c_−1.004LAI+7.054
	*g*_c_, LAI, VPD	267.37^***^	0.228	NEE_r_ = −0.247*g*_c_−1.130LAI+2.214VPD+5.329
	*g*_c_, LAI, VPD, *T*_a_	211.68^***^	0.238	NEE_r_ = −0.254*g*_c_−0.971LAI+3.388VPD−0.155*T*_a_+6.275
May	LAI	570.78^***^	0.180	NEE_r_ = −1.989LAI+8.392
	LAI, *g*_c_	403.46^***^	0.237	NEE_r_ = −1.738LAI−0.268*g*_c_+9.709
	LAI, *g*_c_, VPD	277.50^***^	0.243	NEE_r_ = −1.670LAI−0.264*g*_c_+0.752VPD+8.539
	LAI, *g*_c_, VPD, *T*_a_	214.56^***^	0.249	NEE_r_ = −1.777LAI−0.264*g*_c_+1.788VPD−0.188*T*_a_+11.925

NEE_r_: NEE residual, µmol CO_2_ m^−2^ s^−1^ (the dependent variable);

*g*_c_: canopy conductance, mm s^−1^.

The meanings and units of *T*_a_, VPD, SWC and LAI were the same as Table 2.

Significance of the regression was “^***^” for *P*<0.001.

**Figure 3:**
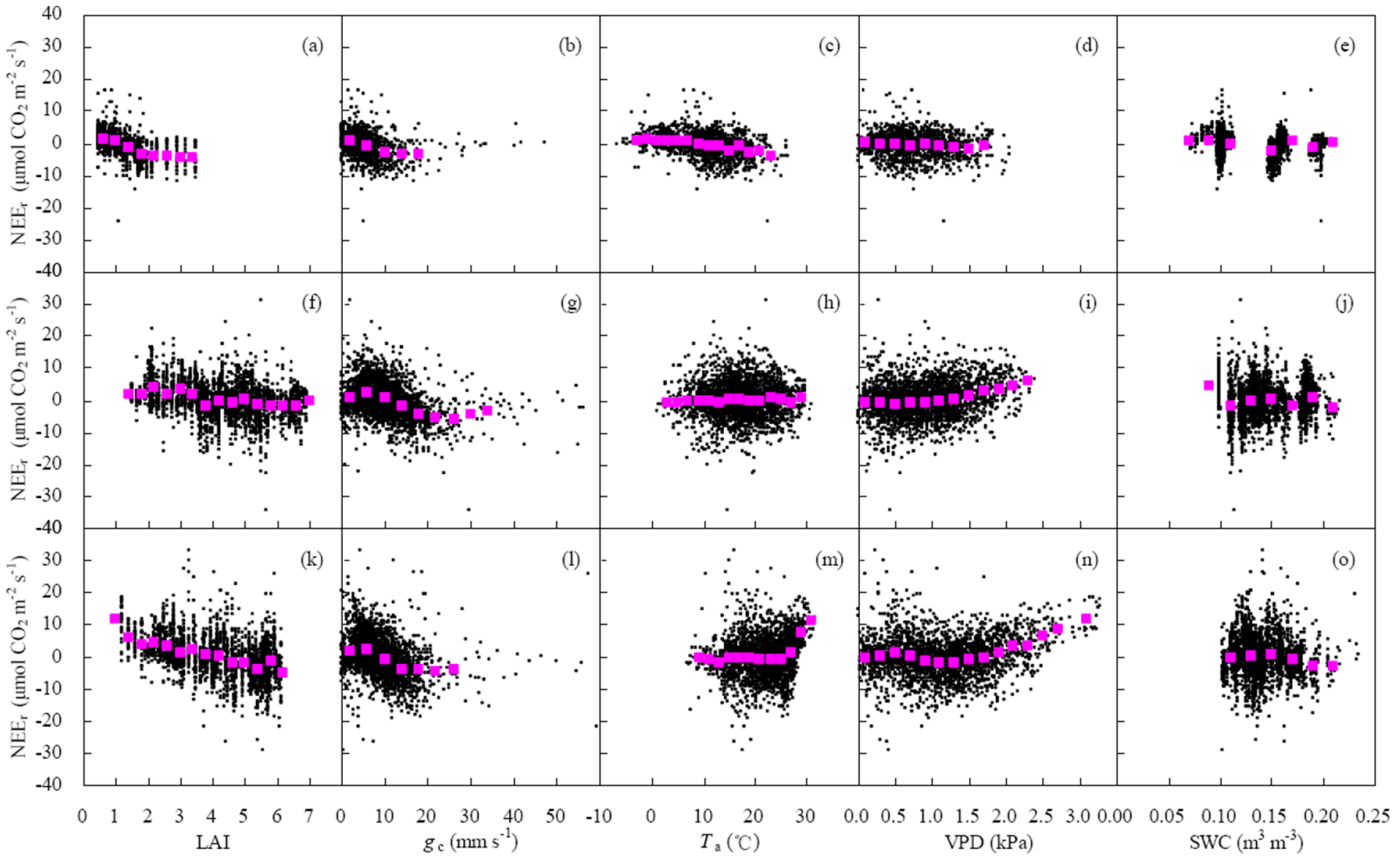
Effects of biophysical factors on NEE residual (NEE_r_) in a winter wheat field in March (a–e), April (f–j) and May (k–o). NEE residual was measured NEE minus simulated NEE. The simulated NEE was obtained by Eq. (3). Biophysical factors include canopy conductance (*g*_c_), Leaf area index (LAI), daytime air temperature (*T*_a_), vapor pressure deficit (VPD) and soil water content at the depth of 20 cm (SWC).

Shifted trends in NEE_r_ versus variables illustrated the limit in linear regression analysis (Fig. 3). With the increase in *T*_a_ and VPD, NEE_r_ turned to increase significantly (*P*<0.001) when *T*_a_ was more than 25°C or VPD more than 1.1−1.3 kPa. With an increase in *g*_c_, the NEE_r_ decline disappeared, or even turned into an increase (*P*<0.01) when *g*_c_ exceed 26 mm s^−1^ in April or 14 mm s^−1^ in March and May (Fig. 3 and Table 4). No reverse trends were found for NEE_r_ versus LAI and SWC.

**Table 4:** Correlation coefficients between NEE residual and influencing variables when the variables were less or more than the thresholds.

Variable	Month	Threshold	Correlation coefficient	
			Variable<Threshold	Variable>Threshold
*T*_a_	May	25°C	−0.006	0.478^***^
VPD	April	1.1 kPa	0.012	0.268^***^
	May	1.3 kPa	−0.133	0.481^***^
*g*_c_	March	14 mm s^−1^	−0.353^***^	0.402^**^
	April	26 mm s^−1^	−0.375^***^	0.029
	May	14 mm s^−1^	−0.283^***^	0.076

Significances of the regression were “^**^” for *P*<0.01 and “^***^” for *P*<0.001.

### Light response parameters: seasonal variation and influencing factors

**Figure 4:**
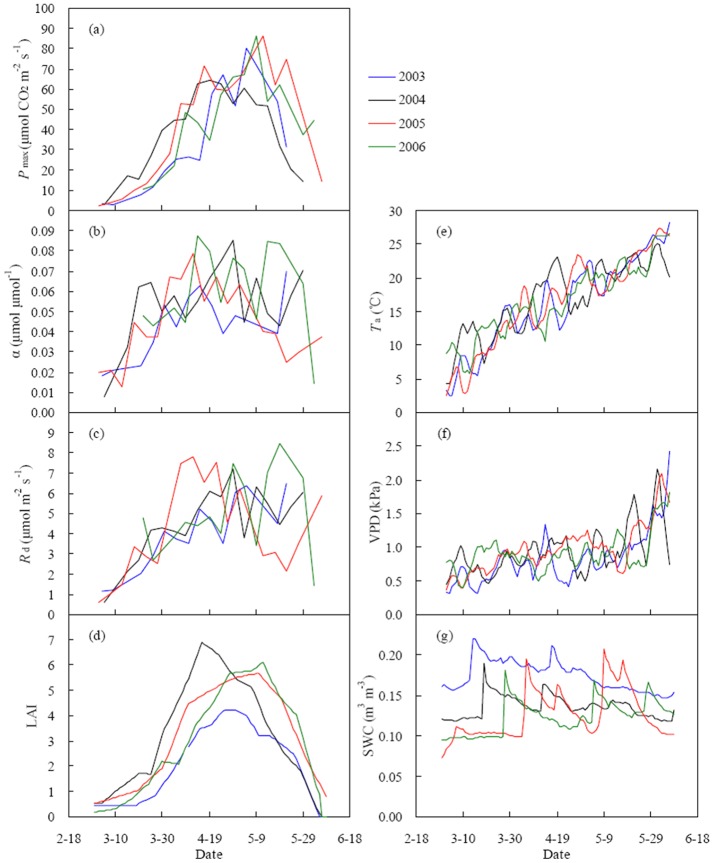
Seasonal variation of initial light use efficiency (α), maximum photosynthetic capacity (*P*_max_), daytime ecosystem respiration under dark conditions (*R*_d_), air temperature (*T*_a_), vapor pressure deficit (VPD) and leaf area index (LAI) in a winter wheat field during the main growing season. α, *P*_max_ and *R*_d_ were obtained at 5-day intervals using a regression model (Eq. (3)).

Light response parameters varied seasonally, with peaks in April or May ranged from 64.2 to 86.1 µmol CO_2_ m^−2^ s^−1^ for *P*_max_, from 0.070 to 0.087 µmol µmol^−1^ for α and from 6.5 to 8.5 µmol CO_2_ m^−2^ s^−1^ for *R*_d_ during the year 2003−2006 (Fig. 4). The seasonal patterns of *P*_max_ were similar with LAI but different from *T*_a_ and VPD which have an increasing tendency from March to May. Compared with other years, *P*_max_ peaked earlier in 2004 when LAI reached the maximum in mid-April due to fast warming in spring (Fig. 4).

**Figure 5:**
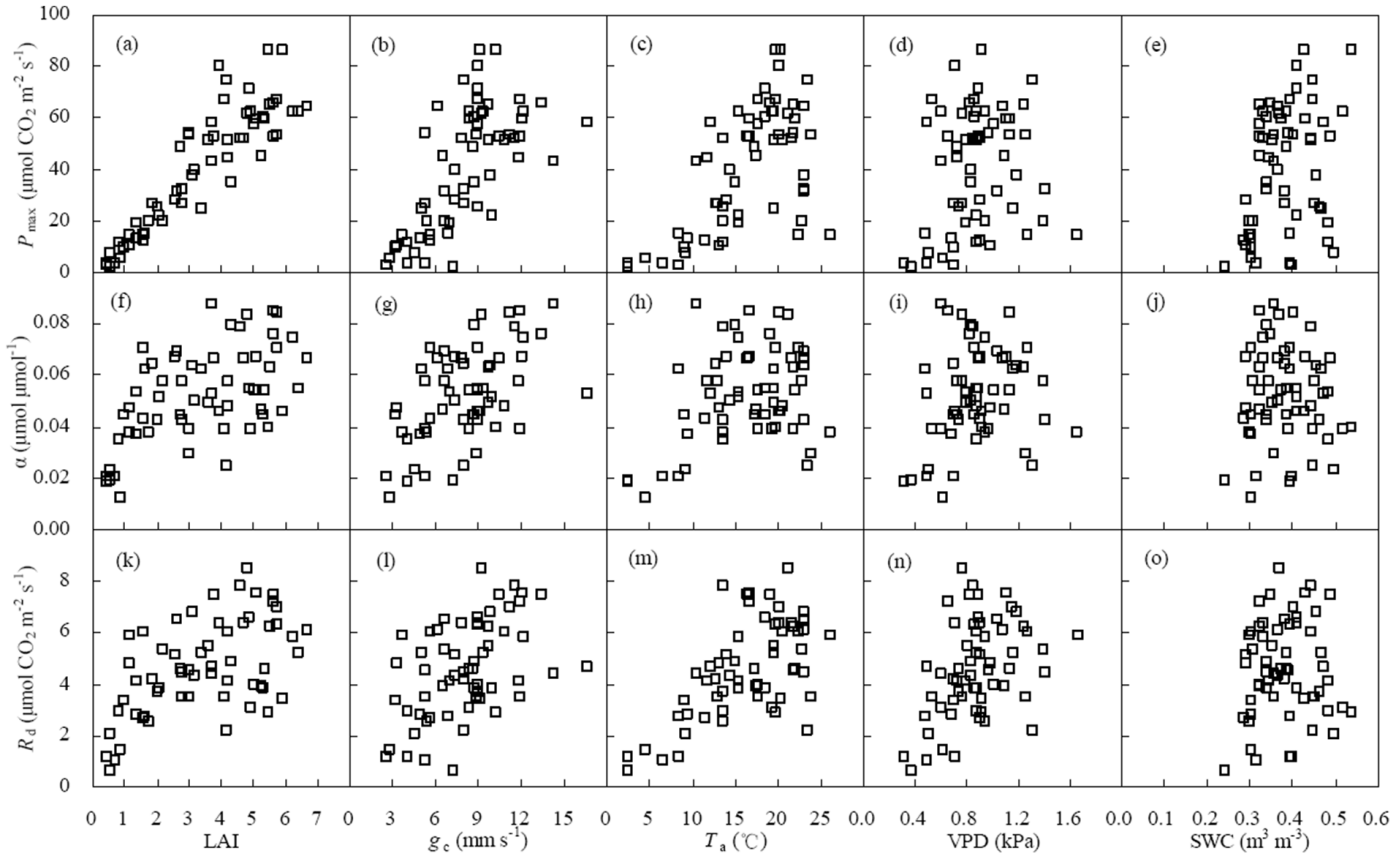
Effects of biophysical factors (*g*_c_, LAI, *T*_a_, VPD and SWC) on light response parameters (α, *P*_max_ and *R*_d_) at 5-day intervals during the main growing season of winter wheat. The meanings of abbreviates were the same as those in Figures 3 and 4.

Figure 5 shows the scattered points of biophysical factors (LAI, *g*_c_, *T*_a_, VPD and SWC) versus NEE light response parameters (α, *P*_max_ and *R*_d_) at 5-day scale during the main growing season of winter wheat. Stepwise multiple linear regression models were used to assess their relationships (Table 5). *P*_max_ increased with the increase in LAI, *g*_c_ and *T*_a_ (*P*<0.001) but reduced with the increase in VPD (*P*<0.001). The factors influencing *P*_max_ were sorted as LAI, *g*_c_, *T*_a_ and VPD. α was proportional to ln(LAI), *g*_c_, *T*_a_ and VPD (*P*<0.001). The impacts of LAI, *g*_c_ and *T*_a_ on α were larger than that of VPD. *R*_d_ was proportional to *T*_a_ (*P*<0.001). It was better to express the relationship between *R*_d_ and *T*_a_ using exponential equation instead of linear equation. However, the influences of SWC on all light response parameters were insignificant (Table 5).

**Table 5:** Effects of biophysical drivers (LAI, *g*_c_, *T*_a_, VPD and SWC) on light response parameters (*P*_max_, α and *R*_d_) at the 5-day scale estimated by the stepwise multiple linear regression models during the growing seasons of 2003–2006.

Dependent variables	Independent variables	*F*	*r* ^2^	Model
*P*_max_	LAI	267.65^***^	0.812	*P*_max_ = 11.8184LAI+1.0615
	LAI, *g*_c_	145.05^***^	0.826	*P*_max_ = 10.3964LAI+1.2911*g*_c_−4.6225
	LAI, *g*_c_, *T*_a_	111.03^***^	0.847	*P*_max_ = 8.9964LAI+1.6064*g*_c_+0.7171*T*_a_−14.4703
	LAI, *g*_c_, *T*_a_, VPD	105.74^***^	0.878	*P*_max_ = 8.1875LAI+1.0694*g*_c_+1.9627*T*_a_−26.0434VPD−4.6112
α	*T*_a_	11.40^**^	0.155	α = 0.0015*T*_a_+0.0273
	*T*_a_, *g*_c_	8.62^***^	0.220	α = 0.0013*T*_a_+0.0019*g*_c_+0.0151
	*T*_a_, *g*_c_, VPD	8.19^***^	0.291	α = −0.0002*T*_a_+0.0030*g*_c_+0.0354VPD−0.0001
	*g*_c_, VPD	12.43^***^	0.290	α = 0.0029*g*_c_+0.0320VPD+0.0005
α	ln(LAI)	14.07^***^	0.185	α = 0.029ln(LAI)+0.040
	ln(LAI), VPD	11.71^***^	0.277	α = 0.026ln(LAI)+0.023VPD+0.021
*R*_d_	*T*_a_	31.98^***^	0.340	*R*_d_ = 0.221*T*_a_+0.897
Ln(*R*_d_)	*T*_a_	32.35^***^	0.343	ln(*R*_d_) = 0.027*T*_a_+0.145

The meanings and units of biophysical factors and light response parameters were the same as Tables 1, 2 and 3.

Significances of the regression were “^**^” for *P*<0.01 and “^***^” for *P*<0.001.

**Figure 6:**
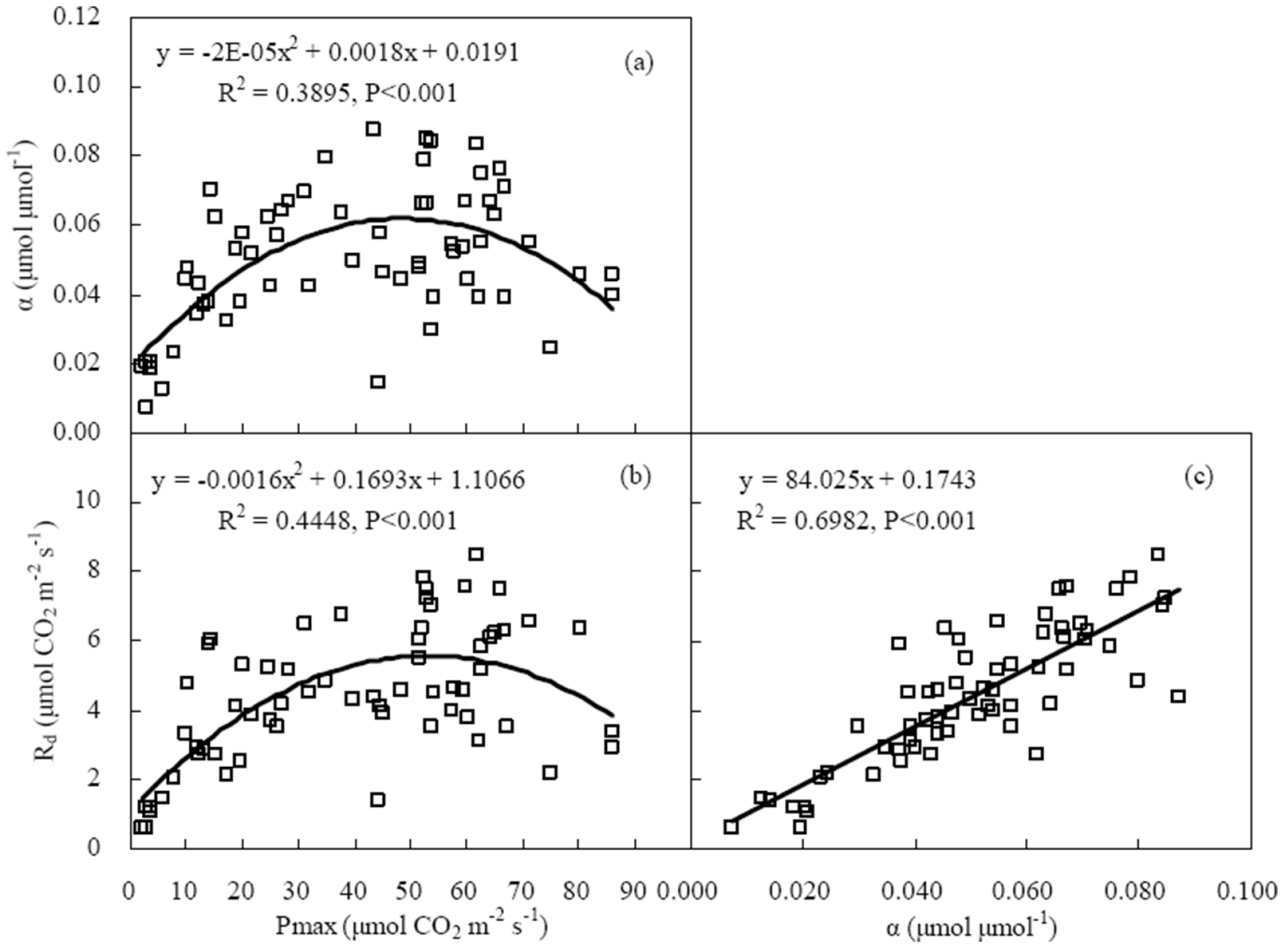
Relationships among initial light use efficiency (α), maximum photosynthetic capacity (*P*_max_), and daytime ecosystem respiration under dark conditions (*R*_d_) at 5-day intervals during the main growing season of winter wheat.

During the growing season of winter wheat, α increased linearly with an increase in *R*_d_ (*P*<0.001) (Fig. 6). α and *R*_d_ enhanced firstly and then declined with the increase in *P*_max_. The maximum α and *R*_d_ appeared when *P*_max_ was around 50 µmol CO_2_ m^−2^ s^−1^. Their relationships could be expressed by quadratic polynomials (*P*<0.001) (Fig. 6).

### NEE Light response and NEE−*g*_c_ relationships under various sky conditions

**Figure 7:**
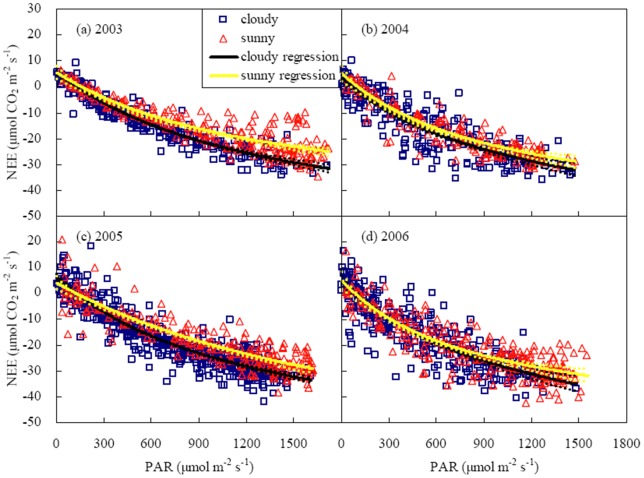
Response of NEE to PAR under sunny and cloudy sky conditions in a winter wheat field during the period of large leaf area index (0.8LAI_max_<LAI<LAI_max_). Light response curves (solid curves) were fitted using the rectangular hyperbolic function (Eq. (3)). The values of light response parameters were shown in Table 3. Between the upper and lower dotted curves were 95% confidence intervals.

Light response curves of NEE were determined under sunny and cloudy sky conditions, respectively (Fig. 7 and Table 6). The analyses were only conducted during the period with large LAI (0.8LAI_max_<LAI<LAI_max_) to limit the influences of crop growth on NEE light response. Compared with sunny sky conditions, *P*_max_ under cloudy conditions was 19%, 12% and 27% higher in 2003, 2004 and 2006 but 14% lower in 2005; α under cloudy skies was 24% and 64% larger in 2003 and 2005, but 5% and 10% less in 2004 and 2006 (Table 6). On average, *P*_max_, α and *R*_d_ under cloudy sky conditions were 9%, 11% and 2% higher than those under sunny sky conditions, respectively. However, owing to large variation in α, *P*_max_ and *R*_d_ among years, their differences between two sky conditions were smaller than their total standard errors calculated by Eq. (4) (Table 7), indicating unremarkable differences in light response parameters between sunny and cloudy sky conditions.

**Table 6:** NEE light response parameters (*P*_max_, α and *R*_d_) derived from Eq.(3) under sunny and cloudy sky conditions in a winter wheat field during the period of large leaf area index (0.8LAI_max_<LAI<LAI_max_).

Year	Skycondition	*P*_max_(µmol CO_2_ m^−2^ s^−1^)	α(µmol µmol^−1^)	*R*_d_(µmol CO_2_ m^−2^ s^−1^)	*r* ^2^	*n*
2003	Sunny	56.0	0.039	5.3	0.812^***^	226
	Cloudy	66.8	0.049	5.4	0.920^***^	302
2004	Sunny	57.5	0.056	5.0	0.839^***^	143
	Cloudy	64.7	0.054	3.2	0.790^***^	261
2005	Sunny	83.0	0.034	3.8	0.799^***^	231
	Cloudy	71.2	0.056	6.1	0.881^***^	567
2006	Sunny	57.2	0.066	4.8	0.747^***^	231
	Cloudy	72.8	0.059	4.5	0.766^***^	325

Significance of the regression was “^***^” for *P*<0.001.

**Table 7:** Comparisons of light response parameters and simulated NEE (NEE_r_) under sunny and cloudy sky conditions.

Items		Skyconditions	Average	Standarderror	Difference(Cloudy-Sunny)	TotalStandard error	Ratio(Cloudy/Sunny)
*P*_max_	(µmol CO_2_ m^−2^ s^−1^)	Sunny	63.42	6.53	5.45	5.95	1.09
		Cloudy	68.87	1.88			
α	(µmol µmol^−1^)	Sunny	0.0489	0.0074	0.0055	0.0068	1.11
		Cloudy	0.0544	0.0022			
*R*_d_	(µmol CO_2_ m^−2^ s^−1^)	Sunny	4.75	0.32	0.08	0.67	1.02
		Cloudy	4.83	0.62			
NEE_s_	(µmol CO_2_ m^−2^ s^−1^)	Sunny	−16.02	1.12	−2.90^*^	1.20	1.18
		Cloudy	−18.92	0.58			

For each year, NEE_r_ was calculated at the same PAR using Eq. (3) and the light response parameters in Table 6. The mean values were obtained for two sky conditions and total standard error was computed using Eq. (4).

The meanings of *P*_max_, α and *R*_d_ were the same as Tables 1.

Significance of the difference was “^*^” for *P*<0.05 if the absolute difference between two sky conditions was greater than the total standard error.

NEE Light response in cloudy days differed from that in sunny days. The differences between two light response curves were significant in 2003 and 2005 but insignificant in 2004 and 2006. Cloudy and sunny light response curves were so close in 2004 and 2006 that their differences were within the confidence intervals due to scattered points of NEE versus PAR (Fig. 7). At the same PAR, simulated net CO_2_ uptake was 33%, 13%, 23% and 8% (averaged 18%) higher under cloudy sky conditions than under sunny sky conditions in 2003, 2004, 2005 and 2006, respectively. The difference between two sky conditions was more than their standard errors (Table 7), suggesting that net CO_2_ uptake under cloudy sky conditions was significantly higher than that under sunny sky conditions.

**Figure 8:**
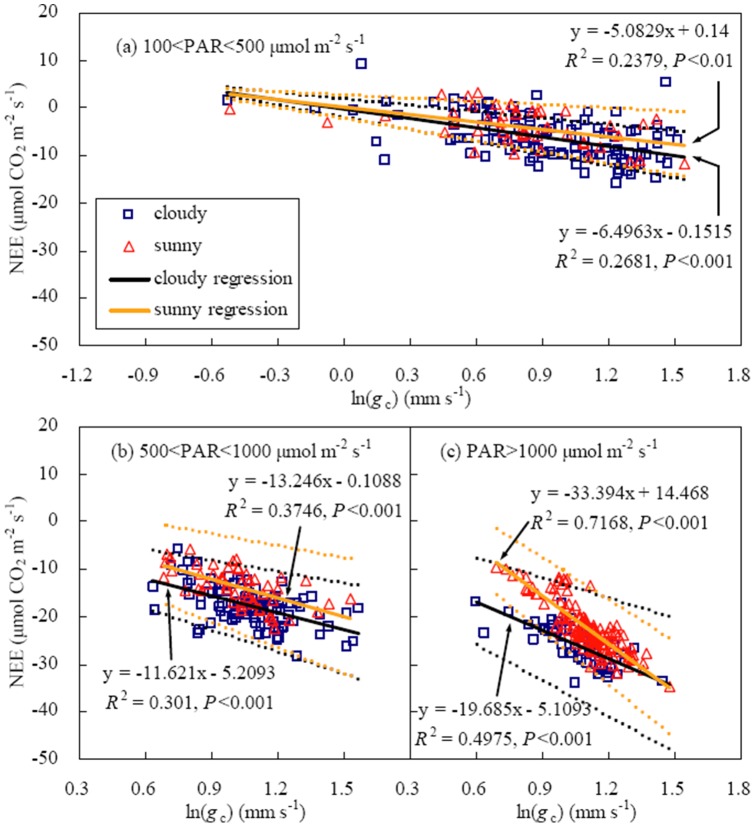
Linear relationship between NEE and logarithmic canopy conductance (*g*_c_) under sunny and cloudy sky conditions (solid lines) in a winter wheat field during the period of large LAI (0.8LAI_max_<LAI<LAI_max_). Between the upper and lower dotted lines were 95% confidence intervals.

As mentioned above, LAI and *g*_c_ were key factors affecting NEE. After neglecting the effects of LAI on NEE during the period with large LAI, the NEE−*g*_c_ relationships were investigated in several radiation classes (Fig. 8). The correlations between NEE and *g*_c_ were described by logarithmic equations under strong, moderate and weak radiation, respectively (*P*<0.001). With the increase in ln(*g*_c_), net CO_2_ uptake enhanced more quickly under strong radiation than under low radiation. At the same *g*_c_, net CO_2_ uptake was higher in cloudy days than in sunny days. Nevertheless, the differences between two regression lines were insignificant for all PAR classes because they were within the wide confidence intervals owing to scattered points of NEE versus ln(*g*_c_) (Fig. 8).

## Discussion

### Factors influencing NEE light response

In boreal and temperate forests, NEE_r_ generally declines with increasing *T*_a_ and VPD. These trends turn to rise when *T*_a_ exceeds 20−25°C [7], [17] and VPD exceeds 1.1−1.3 kPa [7], [18]. In this study, similar phenomena were observed beyond the same thresholds in a temperate cropland (Fig. 3 and Table 4). However, in the tropical forest, NEE_r_ always increases with the increase in *T*_a_ and VPD without reverse trends [40] (Loescher at al., 2003). It may be ascribed to high air temperature and humidity with a narrow range in the tropic region.

Net CO_2_ uptake decreased with the increase in *T*_a_ and VPD (Fig. 3), indicating that temperature and water stress occurred. Under high temperature (*T*_a_>25°C), photosynthesis was prohibited and soil and plant respirations were great, resulting in a lower net CO_2_ uptake than the one simulated by Eq. (3). On the other hand, VPD controls photosynthetic rate through influencing stomatal closure. Under higher VPD, stomatal opening is smaller, leading to the decrease of photosynthetic rate [41]. Moreover, the decline of photosynthetic rate was likely due to low leaf water potential caused by high transpiration rates [42]. Ecosystem respiration is large under higher VPD because of high air and soil temperatures [41]. Hence, net carbon uptake by the cropland dropped. In this study, lower VPD threshold in April than in May (Table 4) implied that wheat plants were more sensitive to the drought at the fast growing stage than at the senescence stage.

Around the critical value, the reverse trends in NEE_r_ versus *g*_c_ (Fig. 3 and Table 4) suggested a shift from stomatal limitation to non-stomatal limitation on daytime net CO_2_ exchange. At dawn, dusk or in cloudy days, photosynthesis was inhibited by weak radiation even though *g*_c_ was large. Higher *g*_c_ threshold in April than in March and May (Table 4) indicated that net CO_2_ uptake was more sensitive to stomatal behavior at the rapid growth stage than at the slow growth senescence stage. Therefore, NEE_r_ was mainly controlled by *g*_c_ instead of LAI in April. However, when *g*_c_ was limited in colder March or drier May, LAI became the most important factor affecting NEE_r_ in these months.

The significant impacts of SWC on NEE_r_ in March (Table 3) may be ascribed to large variation in soil moisture before and after irrigation at the turning green stage of winter wheat (Fig. 4). Nevertheless, the sufficient irrigation concealed these effects in the following two months (Table 3).

### Factors influencing light response parameters

Photosynthetic light response parameters were not constant but varied seasonally [9], [11], [19]. For wheat canopy, mean *P*_max_ in April−May observed in this study (49 µmol CO_2_ m^−2^ s^−1^) was lower than the results (62−67 µmol CO_2_ m^−2^ s^−1^) obtained in a winter wheat field in central Germany [5] and a spring wheat field in Manitoba, Canada [43]. Mean α in April−May obtained in this study (0.056 µmol µmol^−1^) was lower than the value (0.063 µmol µmol^−1^) observed in a winter wheat field in central Germany [5], but higher than that (0.036 µmol µmol^−1^) obtained in a spring wheat field in Manitoba, Canada [43]. The magnitudes of light response parameters varied among different studies might be attributed to the discrepancy in temperature, humidity, canopy structure or/and other factors.

*P*_max_ had positive correlations with LAI [9], [16], [18], [19], air temperature [17], [18] and SWC [9], [18] but negative correlation with VPD [9], [44]. A similar phenomenon was found in this study (Fig. 5 and Table 5). Among all factors, LAI was regarded as a key factor controlling *P*_max_ [9], [16], [18]. *P*_max_ increased linearly [18] or nonlinearly [16], [19] with increasing LAI. The nonlinear relationship was expected as the leaves shade each other in the ecosystem with higher vegetation density [16]. In the cropland, the linear correlation between *P*_max_ and LAI (Fig. 5 and Table 5) illustrated a suitable density range for canopy light intercept.

LAI determines photosynthetic area while *g*_c_ controls the photosynthetic intensity. Owing to the strong link between *g*_c_ and photosynthetic rate, the influences of environmental factors (*T*_a_, VPD and SWC) on photosynthesis may be ascribed to their effects on *g*_c_ [45]. Hence, factors influencing *P*_max_ was sorted by LAI, *g*_c_, *T*_a_ and VPD (Table 5). Our results in an irrigated wheat field differed from those obtained by Zhang et al. [9] who found that *P*_max_ was only affected by LAI and VPD in a dry wheat field.

In this study, α was affected by LAI, *T*_a_, *g*_c_ and VPD in an irrigated wheat field (Table 5), differing from the result reported by Zhang et al. [9] who found that α was only influenced by LAI in a dry wheat field. α represents weak light use efficiency by the plants. At dawn and dusk, the weak light is mainly composed of diffuse radiation. Under low LAI, less diffuse radiation was obtained by the short canopy. When LAI enlarged, the canopy structure was beneficial to receiving scatter light from each direction. Less diffuse radiation was intercepted by the sparse canopy when LAI decreased with leaf senescence. Moreover, at dawn and dusk, low radiation led to small *g*_c_. Under cold and moist conditions, α was more sensitive to *T*_a_ and *g*_c_ than to VPD.

Generally, the key factor influencing respiration was temperature (Table 5). It likely changed to moisture under dry conditions. For instance, Zhang et al. [9] obtained that *R*_d_ had positive correlations with LAI and soil moisture in a dry wheat field. In this study, *R*_d_ was correlated with *T*_a_ significantly but not related to VPD and SWC (Table 5 and Fig. 5) due to sufficient irrigation in the winter wheat field. Furthermore, the effect of LAI on *R*_d_ was unremarkable (Table 5 and Fig. 5) because *R*_d_ was composed of soil and plant respiration and only the later part was related to LAI [16].

After investigating the flux data observed in the grasslands and croplands over the world, Gilmanov et al. [19] pointed out that light response parameters were correlated with each other. Because *P*_max_ was proportional to LAI, α and *R*_d_ correlated to *P*_max_ was the same as correlated to LAI (Table 5, Figs. 5 and 6). Positive correlation between α and *R*_d_ may result from a relatively stable critical PAR (PAR under zero NEE) of 110±6, 99±6, 102±7 and 101±9 µmol m^−2^ s^−1^ in 2003, 2004, 2005 and 2006, respectively.

### NEE Light response and NEE−*g*_c_ relations under various sky conditions

*P*_max_ in a wheat field was greater under cloudy skies than under sunny skies in most of years (Table 6), in agreement with the studies in the temperate forests [8], [21], [22]. Different from the temperate area, Zhang et al. [22] observed a reverse phenomenon in a subtropical forest: net CO_2_ uptake under cloudy skies was less than that under sunny skies. It may be owing to much lower radiation intensity in cloudy days compared with sunny days in the subtropical forest. In the winter wheat field, differences of *P*_max_ and α between two sky conditions were insignificant (Table 6) due to large variations in *P*_max_ and α among years. The values of α in this study were consistent with those reported by Dengel and Grace [8] and Zhang et al. [22] for forests, but different from the results observed by Hollinger et al. [10], Rocha et al. [21] and Suyker et al. [24] who obtained a larger α under cloudy conditions in the forests and an irrigated maize cropland.

In spite of no significant differences in light response parameters between sky conditions, simulated net CO_2_ uptake under cloudy sky conditions was greater than that under sunny sky conditions at the same PAR (Fig. 7). A similar phenomenon was observed in the temperate forests [8], [21] and the irrigated cropland [24]. More CO_2_ uptake by the canopy under cloudy skies than that under sunny skies was owing to many reasons. Firstly, the proportion of diffuse radiation increases under cloudy sky conditions and more light can reach below leaves of the canopies [46]. Photosynthetic rates of shaded leaves are promoted by the delivery of diffuse radiation [47]. In addition, compared with shaded leaves, the phenomenon of saturating photosynthesis easily happens for sunlit leaves because shaded leaves often illuminate brightly [48]. Secondly, canopy conductance was usually higher under cloudy sky conditions than that under sunny sky conditions. Diffuse radiation enhances canopy stomatal conductance mainly due to the reduction in VPD and blue light enrichment within the canopy during cloudy and overcast weather [49], [50]–[52]. Both low VPD [53] and blue light [54], [55] can stimulate stomatal opening and then enhance photosynthetic rate.

In this study, NEE−*g*_c_ relationships were expressed by logarithmic equations (Fig. 8), differing from the result obtained by Dengel and Grace [8] who pointed out that net CO_2_ uptake enhanced linearly with the increase in *g*_c_. Under strong radiation, net CO_2_ uptake enhanced quickly with increasing *g*_c_ (Fig. 8), indicating that *g*_c_ was the main factor affecting NEE. Compared with cloudy days, net CO_2_ uptake was more sensitive to the variation in *g*_c_ for higher temperature and VPD in sunny days.
