# Correction: Biophysical Controls on Light Response of Net CO_2_ Exchange in a Winter Wheat Field in the North China Plain

**DOI:** 10.1371/journal.pone.0101733

**Published:** 2014-07-03

**Authors:** The PLOS ONE Staff

There are multiple errors in Table 7. NEE_r_ should appear as NEE_s_.

Please see the corrected Table 7 here.

The label of the x-axis is missing in Figure 1. Please see the complete, corrected Figure 1 here.

**Figure 1:**
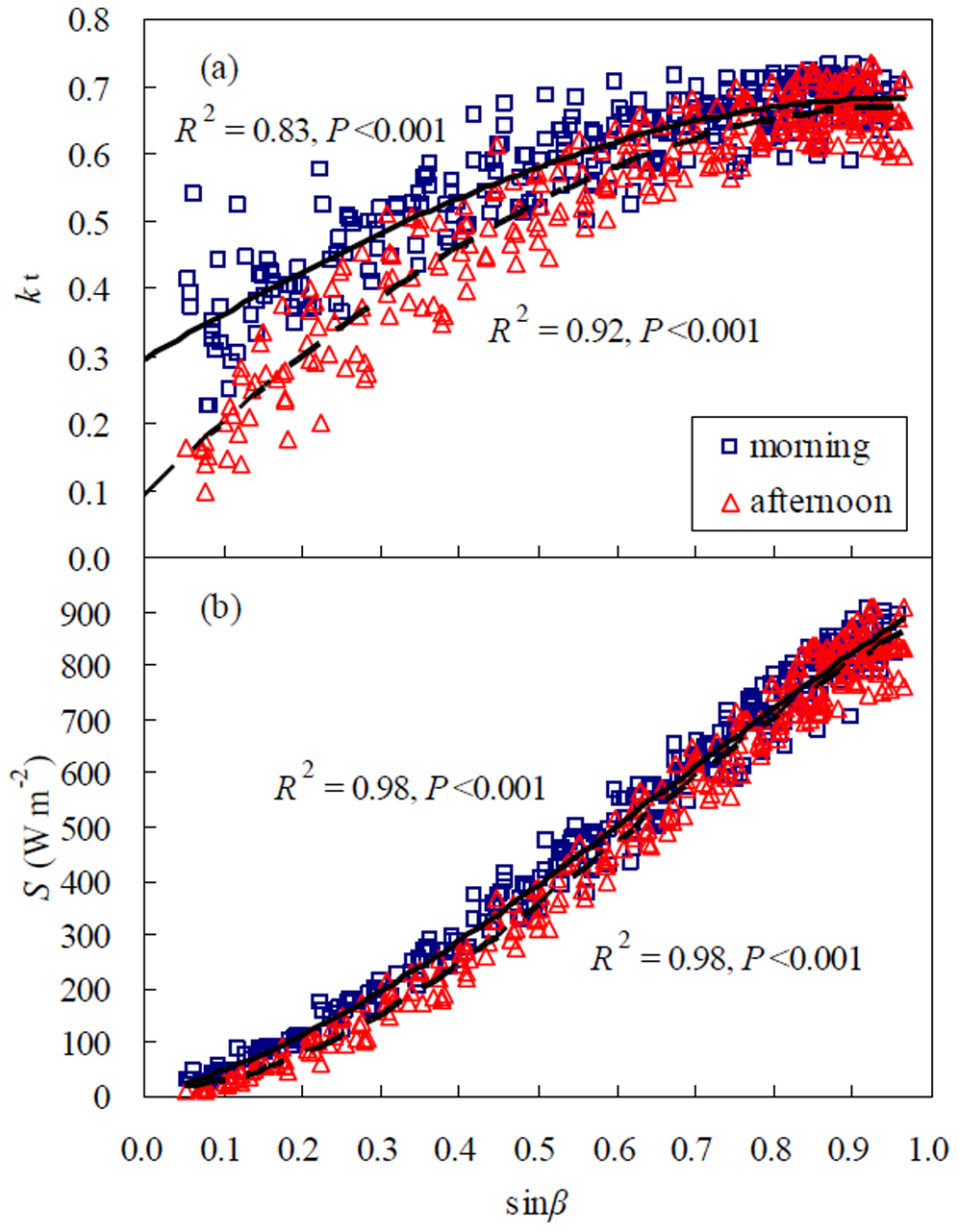
Scatter plots and regressions between (a) the clearness index (*k*_t_) and the sine of solar elevation angles (sin*β*), and (b) global solar radiation (*S*) and sin*β* for a winter wheat field in April-May 2003. The data were fitted by cubic polynomials in the morning (solid line) and afternoon (dashed line), respectively.

**Table 7:** **Comparisons of light response parameters and simulated NEE (NEE_s_) under sunny and cloudy sky conditions.**

Items		Sky conditions	Average	Standard error	Difference (Cloudy-Sunny)	Total Standard error	Ratio (Cloudy/Sunny)
*P*_max_	(μmol CO_2_ m^-2^ s^-1^)	Sunny	63.42	6.53	5.45	5.95	1.09
		Cloudy	68.87	1.88			
α	(μmol μmol^-1^)	Sunny	0.0489	0.0074	0.0055	0.0068	1.11
		Cloudy	0.0544	0.0022			
*R*_d_	(μmol CO_2_ m^-2^ s^-1^)	Sunny	4.75	0.32	0.08	0.67	1.02
		Cloudy	4.83	0.62			
NEE_s_	(μmol CO_2_ m^-2^ s^-1^)	Sunny	-16.02	1.12	-2.90^*^	1.20	1.18
		Cloudy	-18.92	0.58			

For each year, NEE_s_ was calculated at the same PAR using Eq. (3) and the light response parameters in Table 6. The mean values were obtained for two sky conditions and total standard error was computed using Eq. (4).

The meanings of *P*_max_, α and *R*_d_ were the same as Tables 1.

Significance of the difference was “^*^” for *P*<0.05 if the absolute difference between two sky conditions was greater than the total standard error.
